# Identification of candidate genes involved in early iron deficiency chlorosis signaling in soybean (*Glycine max*) roots and leaves

**DOI:** 10.1186/1471-2164-15-702

**Published:** 2014-08-22

**Authors:** Adrienne N Moran Lauter, Gregory A Peiffer, Tengfei Yin, Steven A Whitham, Dianne Cook, Randy C Shoemaker, Michelle A Graham

**Affiliations:** USDA-Agricultural Research Service, Corn Insects and Crop Genetics Research Unit, 1565 Agronomy Hall, Ames, IA 50011 USA; Department of Statistics, Iowa State University, Ames, Iowa 50011 USA; Department of Plant Pathology and Microbiology, Iowa State University, Ames, Iowa 50011 USA; Department of Agronomy, Iowa State University, Ames, Iowa 50011 USA

**Keywords:** Iron deficiency chlorosis, Soybean, *Glycine max*, RNA-Seq

## Abstract

**Background:**

Iron is an essential micronutrient for all living things, required in plants for photosynthesis, respiration and metabolism. A lack of bioavailable iron in soil leads to iron deficiency chlorosis (IDC), causing a reduction in photosynthesis and interveinal yellowing of leaves. Soybeans (*Glycine max* (L.) Merr.) grown in high pH soils often suffer from IDC, resulting in substantial yield losses. Iron efficient soybean cultivars maintain photosynthesis and have higher yields under IDC-promoting conditions than inefficient cultivars.

**Results:**

To capture signaling between roots and leaves and identify genes acting early in the iron efficient cultivar Clark, we conducted a RNA-Seq study at one and six hours after replacing iron sufficient hydroponic media (100 μM iron(III) nitrate nonahydrate) with iron deficient media (50 μM iron(III) nitrate nonahydrate). At one hour of iron stress, few genes were differentially expressed in leaves but many were already changing expression in roots. By six hours, more genes were differentially expressed in the leaves, and a massive shift was observed in the direction of gene expression in both roots and leaves. Further, there was little overlap in differentially expressed genes identified in each tissue and time point.

**Conclusions:**

Genes involved in hormone signaling, regulation of DNA replication and iron uptake utilization are key aspects of the early iron-efficiency response. We observed dynamic gene expression differences between roots and leaves, suggesting the involvement of many transcription factors in eliciting rapid changes in gene expression. In roots, genes involved iron uptake and development of Casparian strips were induced one hour after iron stress. In leaves, genes involved in DNA replication and sugar signaling responded to iron deficiency. The differentially expressed genes (DEGs) and signaling components identified here represent new targets for soybean improvement.

**Electronic supplementary material:**

The online version of this article (doi:10.1186/1471-2164-15-702) contains supplementary material, which is available to authorized users.

## Background

Iron is an important micronutrient for all living things. In plants, it is essential for photosynthesis, respiration and other metabolic processes. Plants adjust their iron uptake from the soil to achieve the proper cellular iron concentrations. Without enough iron, plants suffer Iron Deficiency Chlorosis (IDC), which is among the most common and potentially severe nutritional stresses in plants [1]. IDC is typically not due to low amounts of iron in the soil, but rather to an unusable ferric (Fe^3+^) state. Too much iron is also problematic, as free iron leads to reactive oxygen species (ROS), DNA damage, and other cellular stress [2]. Therefore, iron homeostasis is tightly controlled by regulating iron uptake, transport and storage. IDC is a global problem, but is especially problematic in the calcareous soils of the Midwestern U.S., where the majority of U.S. soybeans are grown [3]. Calcareous soil is identified by the presence of calcium carbonate (or lime) and a pH higher than 7, which keeps iron in the ferric (Fe^3+^) state. Many high yielding soybeans are susceptible to IDC, which results in a yellowing of leaves due to reduced photosynthesis. Symptoms vary in degree of severity, but can result in total yield loss. The recommended management for IDC is growing IDC-resistant soybean lines. However, resistant lines yield lower than susceptible lines in conditions that do not favor the development of IDC. Identifying the genetic basis of IDC resistance may aid in the development of IDC tolerant lines that perform well in multiple soil types.

Plants have two mechanisms for acquiring iron from the soil (Strategy I and II). Strategy I, which occurs in most dicots including soybean, functions through the induction of the *Fe-deficiency Induced Transcription Factor* (*FIT*) in the root, which regulates *Ferric-chelate Reductase* (*FRO*) and *Iron-Regulated Transporter* (*IRT)* [4–6]. While these genes are activated in the root, it is believed that the signal activating these genes comes from an unknown factor that originates in the leaves [7, 8]. Hormones are obvious candidates for controlling signaling from the shoot to the root. Garcia *et al*. [9] demonstrated that the hormones ethylene and nitric oxide act early in response to IDC in the roots and are necessary for the induction of iron uptake genes. Thus far, the study of ethylene and nitric oxide function has been limited to the roots. Examining the gene expression in multiple tissues during IDC response may allow for the construction of a complete signaling pathway.

In the early 1970’s, near isogenic soybean lines (NILs), Clark (PI54833) and Isoclark, were developed that differ in their responses to iron stress [10]. The cultivar Clark (PI54833) is iron efficient and does not develop IDC symptoms in iron-limiting conditions. Isoclark is susceptible to iron stress and develops interveinal chlorosis in response to iron limitation. Gene expression comparisons between Clark and Isoclark, which share 98% genetic identity, have allowed the identification of hundreds of genes involved in iron stress responses in soybean. In the last several years, microarray analyses, RNA-Seq, introgression and association mapping, sub-NIL development and virus-induced gene silencing (VIGS) have been used to identify and characterize soybean genes that are differentially expressed during iron stress and iron stress recovery [11–15]. However, the early signaling events in the iron efficiency stress response remain elusive. Therefore, the work presented here aims to capture early transcriptional responses to iron stress in the iron efficient line Clark. We have used RNA-Seq to measure transcriptional responses one and six hours after iron stress. The differentially expressed genes (DEGs) and signaling components identified here represent new targets for soybean improvement.

## Results

### RNA-Seq reveals a dynamic change in gene expression in response to IDC

In order to identify early responses to iron stress, we quantified expression of genes at one and six hours post iron stress in leaves and roots of the iron efficient line Clark. While other studies have used both Clark and Isoclark, previous work [13] has demonstrated that Isoclark is iron inefficient and does not induce expression of genes involved in iron homeostasis in response to iron stress. Therefore, we limited our study to Clark. To induce iron stress, the roots of plants grown in iron-sufficient (100 μM Fe(NO3)3•9H2O) conditions for sixteen days were rinsed in distilled water and then plants were transferred into either iron-sufficient (100 μM Fe(NO3)3•9H2O) or iron-deficient (50 μM Fe(NO3)3•9H2O) conditions. Two replicates of root and 1st trifoliate leaf tissues were collected at one and six hours after transfer to sufficient or insufficient conditions for a total of eight sample types. Following RNA isolation, samples were sent to the National Center for Genomic Research for single-end RNA sequencing on an Illumina Genome Analyzer II with a read length of 36 bp. Following the bioinformatic pipeline detailed in the Materials and Methods, a total of 507,784,149 reads (252,907,004 from 8 leaf samples, 254,877,145 from 8 root samples) were mapped to the soybean genome (*G. max* version 1.1 [16]). The Illumina reads generated by this study were deposited in the National Center for Biotechnology Short Read Archive (NCBI SRA Bioproject accession SRP031889).

**Figure 1:**
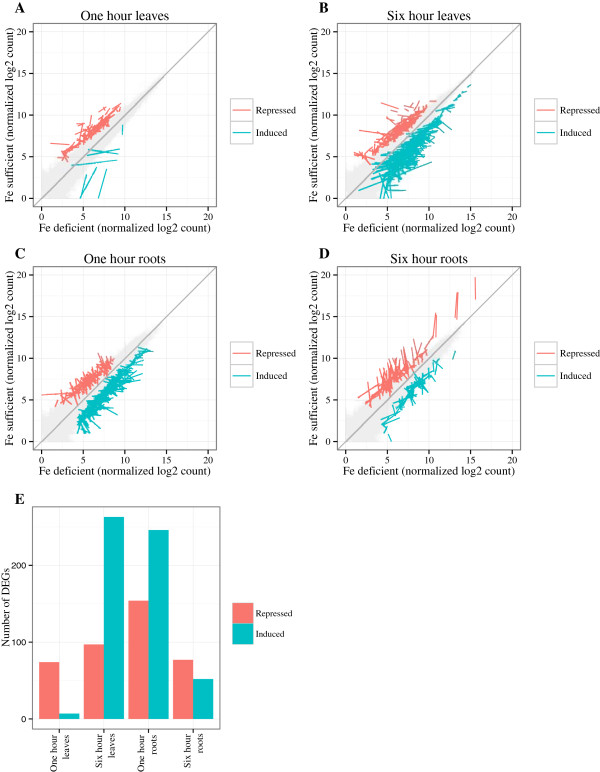
**Genes significantly differentially expressed in response to iron stress.** Significantly differentially expressed genes (DEGs) (FDR < 0.05) were identified by comparing gene expression in iron deficient conditions to iron sufficient conditions (D/S). Porcupine plots were used to visualize the expression of all genes and all DEGs. Expression of all genes is shown in grey. Expression of DEGs is shown in red (repressed by iron deficiency) and blue (induced by iron deficiency). A line joins replicates of DEGs. **A**. DEGs from leaves after one hour of iron stress. **B**. DEGs from leaves after six hours of iron stress. **C**. DEGs from roots after one hour of iron stress. **D**. DEGs from roots after six hours of iron stress. **E**. Bar graph of the total number of repressed or induced differentially expressed genes at each tissue and time point.

To identify genes differentially expressed in response to iron stress in each sample, we used the edgeR [17] statistical package comparing deficient and sufficient replicates at a given time point and tissue. Most RNA-Seq analysis tools provide a list of DEGs and report average expression across replicates. However, one bad replicate can extremely bias which genes are identified as differentially expressed and the level and direction of expression. Therefore, it is important to use statistical packages that report expression of all replicates and use visualization tools of raw and normalized data to verify biological and technical reproducibility of replicates. This step is particularly important in experiments such as ours, where only two replicates were used. We used the package ggplot2 (CRAN, [18]) to compare normalized gene expression in replicate data sets. In addition, ggplot2 was used to create porcupine plots [19] of significantly differentially expressed genes at multiple False Discovery Rates (FDR) (Figure 1, Additional file 1) relative to the expression of all genes. The porcupine plots use lines to connect replicate data points, allowing visual identification of any problematic data. Following these analyses, genes were considered significant if they had a fold change > 2.0 (deficient/sufficient) and a false discovery rate (FDR) <0.05. The DEGs were annotated using the SoyBase Genome Annotation Report page (http://soybase.org/genomeannotation/index.php), which provided UniRef100 [20] hit information, best *A. thaliana* homologs and gene ontology information inferred from *A. thaliana* (The Arabidopsis Information Resource [TAIR] version 10, http://www.arabidopsis.org). The DEGs and their annotations are provided for each tissue and time point (Additional files 2, 3, 4 and 5).

We identified 81 and 400 DEGs in response to iron stress in one hour and six hour leaves, respectively, and 360 and 129 DEGs were identified in one hour and six hour roots (Figure 1). Surprisingly, there was little overlap in the DEGs identified within the same tissue at different time points or across tissues at the same time point. Only seven genes were in common between one and six hour leaves, and another four genes were in common between one and six hour roots (Additional files 2, 3, 4 and 5). For nine of these eleven genes, the direction of expression changed between the one hour and six hour time points. Similarly, only eleven genes were in common between roots and leaves, regardless of time point. The small degree of overlap in DEGs across sample types accompanied by large changes in expression levels between time points suggests dynamic and distinct responses to iron stress occur in leaves and roots.

To develop an understanding of which genes were affected by iron stress, we began focusing on the top ten induced and repressed genes in each sample, paying particular attention to those genes with homology to Arabidopsis genes with known roles in nutrient stress responses, growth and signaling (Table 1). In one hour leaves, iron deficiency led to decreased expression of 74 of the 81 DEGs. The seven genes induced by iron deficiency included homologs of previously described Arabidopsis genes *AtPLP1* (Glyma07g13790, 6E^-89^), *AtGASA1* (Glyma14g40400, 4E^-32^), *AtSWEET12* (Glyma05g38351, 1E^-53^), two homologs of *AtOXS3* (Glyma11g33040 [2E^-15^] and Glyma18g05160 [6E^-16^]) and a copper amine oxidase (Glyma01g07860 [E = 0]) (Table 1). *AtPLP1* is patatin-related phospholipase that is differentially expressed in response to phosphate stress [21]. *AtGASA1* (GA-Stimulated in Arabidopsis) is a gibberellic acid-regulated protein and expressed in rosettes [22]. *AtSWEET12* is a sucrose transporter involved in phloem loading that transfers sucrose from the leaves to nonphotosynthetic tissues elsewhere in the plant [23]. Overexpression of *AtOXS3* in Arabidopsis resulted in greater tolerance to heavy metals and oxidative stress. Copper amine oxidase is upregulated in response to wounding in chickpea [24] and in response to nematode infection but not wounding, in Arabidopsis [25]. The top ten genes repressed by iron deficiency included two homologs of *AtNIA1* (Glyma06g11430 [E = 0] and Glyma13g02510 [E = 0]) and a homolog of *AtDXR* (Glyma16g10880, E = 0). *AtNIA* is required for nitric oxide (NO) production [26]. NO acts an important signaling molecule for a variety of abiotic stresses including iron deficiency and drought [27]. *AtDXR* is localized to the chloroplast and catalyzes the first committed step of isoprenoid biosynthesis leading to the production of chlorophyll, carotenoids, ABA, brassinosteroids, cytokinins and gibberellins [28].

**Table 1:** **Top ten significantly induced and repressed DEGs under iron stress at each time and tissue**

Leaf one hour			
Glyma 1.1 ID	Log _2_fold change	TAIR10 annotation	E-value
Glyma11g12650	5.00	NA	NA
Glyma07g13790	3.78	PLP1, AtPLAIVA | Acyl transferase/hydrolase/lysophospholipase superfamily protein	6.0E-89
Glyma14g40400	3.37	GASA1 | GAST1 protein homolog 1	4.0E-32
Glyma05g38351	3.06	MTN3, SWEET12, AtSWEET12 | homolog of Medicago truncatula	1.0E-53
Glyma11g33040	2.83	OXS3, ATOXS3 | oxidative stress 3	2.0E-15
Glyma18g05160	2.59	OXS3, ATOXS3 | oxidative stress 3	6.0E-16
Glyma01g07860	1.28	Copper amine oxidase family protein	0.0E + 00
Glyma01g35620	-4.38	Long-chain fatty alcohol dehydrogenase family protein	0.0E + 00
Glyma09g35210	-4.05	Long-chain fatty alcohol dehydrogenase family protein	0.0E + 00
Glyma18g38410	-4.05	MuDR family transposase	2.0E-44
Glyma16g10880	-3.78	DXR, PDE129 | 1-deoxy-D-xylulose 5-phosphate reductoisomerase	0.0E + 00
Glyma16g15790	-3.61	WEB1 | Plant protein of unknown function (DUF827)	4.0E-170
Glyma13g02510	-3.36	NIA1, GNR1, NR1 | nitrate reductase 1	0.0E + 00
Glyma06g11430	-3.24	NIA1, GNR1, NR1 | nitrate reductase 1	0.0E + 00
Glyma13g39440	-2.83	CER4, G7, FAR3 | Jojoba acyl CoA reductase-related male sterility protein	0.0E + 00
Glyma01g25890	-2.80	Major facilitator superfamily protein	0.0E + 00
Glyma07g37380	-2.77	Protein phosphatase 2C family protein	1.0E-164
**Leaf six hours**			
**Glyma 1.1 ID**	**Log**_**2**_ **fold change**	**TAIR10 annotation**	**E-value**
Glyma15g18360	6.37	XTR6, XTH23 | xyloglucan endotransglycosylase 6	3.0E-141
Glyma02g39320	5.94	ASN1, DIN6, AT-ASN1 | glutamine-dependent asparagine synthase 1	0.0E + 00
Glyma03g37970	4.63	ATGPAT2, GPAT2 | glycerol-3-phosphate acyltransferase 2	0.0E + 00
Glyma09g24170	4.18	Heavy metal transport/detoxification superfamily protein	2.0E-19
Glyma11g27480	3.81	ASN1, DIN6, AT-ASN1 | glutamine-dependent asparagine synthase 1	0.0E + 00
Glyma06g08540	3.80	RD22, ATRD22 | BURP domain-containing protein	4.0E-124
Glyma08g45281	3.77	NA	NA
Glyma01g32450	3.70	WNK5 | with no lysine (K) kinase 5	0.0E + 00
Glyma03g37990	3.61	ATGPAT2, GPAT2 | glycerol-3-phosphate acyltransferase 2	0.0E + 00
Glyma16g21050	3.59	ABCG14 | ATP-binding cassette 14	0.0E + 00
Glyma14g35340	-5.53	EXO | Phosphate-responsive 1 family protein	2.0E-146
Glyma14g35330	-4.69	EXO | Phosphate-responsive 1 family protein	5.0E-158
Glyma01g01500	-4.47	Mono-/di-acylglycerol lipase, N-terminal;Lipase, class 3	1.0E-80
Glyma01g01530	-4.12	Mono-/di-acylglycerol lipase, N-terminal;Lipase, class 3	1.0E-10
Glyma11g03500	-4.08	Eukaryotic aspartyl protease family protein	1.0E-170
Glyma13g33780	-3.90	NA	NA
Glyma16g01430	-3.59	SAUR-like auxin-responsive protein family	6.0E-37
Glyma02g38200	-3.58	Octicosapeptide/Phox/Bem1p family protein	2.0E-49
Glyma06g10710	-3.35	EXO | Phosphate-responsive 1 family protein	9.0E-154
Glyma02g06810	-3.33	Unknown protein	1.0E-37
**Root one hour**			
**Glyma 1.1 ID**	**Log**_**2**_ **fold change**	**TAIR10 annotation**	**E-value**
Glyma10g02730	4.85	RCI3, RCI3A | Peroxidase superfamily protein	9.0E-120
Glyma17g27187	4.66	Kinase interacting (KIP1-like) family protein (NET1D)	2.0E-139
Glyma03g28850	3.90	BG1 | beta-1,3-glucanase 1	1.0E-138
Glyma02g17060	3.85	RCI3, RCI3A | Peroxidase superfamily protein	3.0E-118
Glyma17g23660	3.77	Kinase interacting (KIP1-like) family protein (NET1D)	1.0E-140
Glyma19g31580	3.67	BG1 | beta-1,3-glucanase 1	3.0E-137
Glyma15g12600	3.64	Bifunctional inhibitor/lipid-transfer protein/seed storage 2S albumin superfamily protein	1.0E-35
Glyma17g27135	3.61	Kinase interacting (KIP1-like) family protein (NET1D)	7.0E-147
Glyma03g02834	3.40	NA	NA
Glyma09g01680	3.16	Bifunctional inhibitor/lipid-transfer protein/seed storage 2S albumin superfamily protein	2.0E-34
Glyma05g02040	-3.46	NA	NA
Glyma08g27660	-3.25	ATMYB121, MYB121 | myb domain protein 121	2.0E-62
Glyma18g38410	-2.86	MuDR family transposase	2.0E-44
Glyma10g41670	-2.83	NA	NA
Glyma11g05517	-2.58	NA	NA
Glyma06g05990	-2.50	Protein kinase superfamily protein	0.0E + 00
Glyma10g28850	-2.35	Unknown protein	1.0E-48
Glyma07g18280	-2.30	2-oxoglutarate (2OG) and Fe(II)-dependent oxygenase superfamily protein	4.0E-170
Glyma20g23020	-2.27	Unknown protein	1.0E-49
Glyma04g17300	-2.23	NA	NA
**Root six hours**			
**Glyma 1.1 ID**	**Log**_**2**_ **fold change**	**TAIR10 annotation**	**E-value**
Glyma19g41920	5.74	NA	NA
Glyma13g37770	3.60	Wound-responsive family protein	3.0E-17
Glyma03g39341	3.45	AtPP2-B15, PP2-B15 | phloem protein 2-B15	1.0E-18
Glyma15g10693	3.40	Protein kinase superfamily protein	2.0E-78
Glyma20g00604	3.14	ATOMT1, OMT1 | O-methyltransferase 1	6.0E-53
Glyma01g06774	3.10	ATBOR4, BOR4 | HCO3- transporter family	2.0E-20
Glyma05g36310	2.90	ACO1, ATACO1 | ACC oxidase 1	2.0E-159
Glyma14g39910	2.84	Prolyl oligopeptidase family protein	0.0E + 00
Glyma13g10791	2.47	ZIP1 | zinc transporter 1 precursor	5.0E-129
Glyma18g41760	2.36	Proton pump interactor 1	2.0E-45
Glyma05g09990	-4.03	NA	NA
Glyma16g29233	-3.96	NA	NA
Glyma16g29216	-3.89	Disease resistance family protein/LRR family protein	5.0E-93
Glyma05g16286	-3.86	NA	NA
Glyma13g12815	-3.85	NA	NA
Glyma15g03080	-3.55	NA	NA
Glyma04g33460	-3.54	NA	NA
Glyma01g04545	-3.53	NA	NA
Glyma01g04545	-3.54	NA	NA
Glyma09g24780	-3.51	NA	NA

The top and bottom ten genes significantly (FDR < 0.05) differentially expressed at each time and tissue under iron stress. Glyma1.1 ID refers to *Glycine max* version 1.1 release. A positive log_2_ fold change represents induction in response to iron deficiency while a negative fold change represents repression in response to iron deficiency. The top *A. thaliana* hit (TAIR version 10) was determined by BLASTP [52] of Glyma1.1 primary proteins against *A. thaliana* proteins (TAIR10, E < 10^-6^). DEGs with no BLASTP hit to *A. thaliana* are indicated by NA (not applicable). Full annotation information can be found in Additional files 2, 3, 4 and 5.

In one hour roots, 263 of the 360 DEGs were induced in response to iron stress. The top ten induced genes included two homologs of *AtRCI3* (Glyma10g02730 [9E^-120^] and Glyma02g17060 [3E^-118^]), two homologs of *AtBG1* (Glyma03g28850 [1E^-138^] and Glyma19g31580 [3E^-137^]), and three homologs of *NET1D* (Glyma17g27187 [2E^-139^], Glyma17g27135 [7E^-147^] and Glyma17g23660 [1E^-140^]). *AtRCI3* (Rare Cold Inducible gene 3) is a peroxidase that is involved in salt-tolerance, dehydration and potassium deficiency signaling [29, 30]. BG1 responds to a variety of biotic stresses in Arabidopsis [31]. NET1D is an actin-binding protein highly expressed in the stele and conducting tissues of the roots [32]. Genes repressed by iron deficiency included a homolog of 2OG-Fe(II)-dependent oxygenase superfamily protein (Glyma07g18280 [4E^-170^]) and *AtMYB121* (Glyma08g27660 [2E^-62^]). 2OG-Fe(II)-dependent oxygenase family members are involved in hormone synthesis in plants, particularly ethylene synthesis [33]. *AtMYB121* responds to salinity stress in Arabidopsis roots [34].

Six hours after plants were transferred from iron sufficient to iron deficient media, iron deficiency induced the expression of 246 genes in leaves but repressed the expression of 154. Induced genes included a homolog of *AtRD22* (Glyma06g08540 [4E^-124^]), *WNK5* (Glyma01g32450 [E = 0]) and two homologs of *ASN1/AtDIN6* (Glyma02g39320 [E = 0] and Glyma11g27480 [E = 0]). Arabidopsis *AtRD22* is responsive to abscisic acid, water and salt stress [35]. The rice homolog of WNK5 (with no lysine kinase), *OsWNK1*, has a suspected role in abiotic stress tolerance and is involved in circadian rhythm [36]. The wheat homolog of asparagine synthetase *TaASN1* has been shown to be upregulated in roots in response to salt, drought and ABA stress [37]. The top ten genes repressed by iron deficiency included three homologs of *AtEXO* (Glyma14g35340 [2E^-146^], Glyma14g35330 [5E^-158^] and Glyma06g10710 [9E^-154^]). The extracellular EXO protein is essential for cell expansion and promotes shoot and root growth [38]. *AtEXO* mutants have altered expression of sugar-responsive genes and increased ABA levels. It is interesting to note that eight EXO and EXO-like (EXL5) homologs were differentially expressed in response to iron stress at this time point.

In six hour roots, 52 genes were upregulated in response to iron deficient conditions while 77 genes were downregulated. The top ten genes induced in response to iron deficiency included a homolog of a wound-responsive family member (Glyma13g37760 [1E^-29^]), *AtOMT1* (Glyma20g00604 [6E^-53^]), *AtBOR4* (Glyma01g06774 [2E^-20^]), *AtACO1* (Glyma05g36310 [2E^-159^]) and *ZIP1* (Glyma13g10791 [5E^-129^]). *AtOMT1* is involved in lignin formation and the biosynthesis of sinapate esters [39]. *AtBOR4* (Borate efflux transporter 4) overexpression in rice increased tolerance to excess boron [40]. Trafficking of *AtBOR4* to the outer polar domain defines the root-soil interface [41]. *AtACO1* (ACC oxidase 1) is an ethylene biosynthetic gene [42, 43]. *AtZIP1* functions as a zinc transporter, and is upregulated in *AtIRT1* knockouts [44]. Interestingly, IRT1 and IRT2 are both ZIP family members. *AtZIP1* appears to function in both iron and zinc homeostasis. Of the top ten genes repressed by iron stress in six hour roots, only one has an identified homolog in Arabidopsis, the function of which was unknown.

### DEGs located within introgressed regions associated with iron inefficiency

Recently, introgression mapping was used to identify regions introgressed from the iron inefficient donor parent T203 to the iron efficient line Clark to develop Isoclark (iron inefficient). Collectively, Severin *et al*. [45] and Stec *et al.* [46] identified introgressed regions on soybean chromosomes 3, 4, 5, 8, 13 and 16 of Isoclark. Several studies have identified quantitative trait loci (QTL) for iron deficiency in soybean [15, 47–49]. However, only the studies by Mamidi *et al.* [49] and Lin *et al.* [47, 48] identified QTL on the same chromosomes as the introgressed regions. By comparing the sequences of the molecular markers used in these studies to the introgressed regions, only the QTL identified on chromosome 3 [47–49] corresponded to an introgressed region (data not shown). Five DEGs corresponded to the region on chromosome 3 [14]. In six hour leaves we identified a S-adenosyl-L-methionine-dependent methyltransferases superfamily protein (Glyma03g28490). From one hour roots we identified a βHLH038 homolog (Glyma03g28630), an ethylene responsive binding factor (*AtERF15*, Glyma03g31940) and a disease resistance-responsive protein (Glyma03g30360). Glyma03g28630 was recently identified as one of 12 candidate genes underlying the IDC QTL on soybean chromosome 3 by Peiffer *et al.* [14]. From six hour roots we identified a differentially expressed ethylene response factor (Glyma03g31940).

It is important to note that complex traits, such as IDC, can be the result of a number of small genes with minor effects. Therefore, it is worth noting the DEGs located within introgressed regions but not associated with a QTL. In six hour leaves, we identified a cell division control 6 ortholog (Glyma08g45230) and a sucrose–proton symporter (*AtSUC2*, Glyma16g27350). In roots at one hour, there were six DEGs of interest from introgressed regions including homologs of growth regulating factor 4 (*AtGRF4*, Glyma03g35010), expansin A7 (*AtEXPA17*, Glyma03g38480), Late embryogenesis abundant protein (*AtNHL10*, Glyma03g35920 and Glyma03g35980), flavin-binding monooxygenase protein (Glyma05g35430), and a glycosyl hydrolase (Glyma05g34850). In six hour roots, we identified a homolog of RNA-binding family protein (Glyma08g44150).

### Plant pathways responding to iron stress

**Figure 2:**
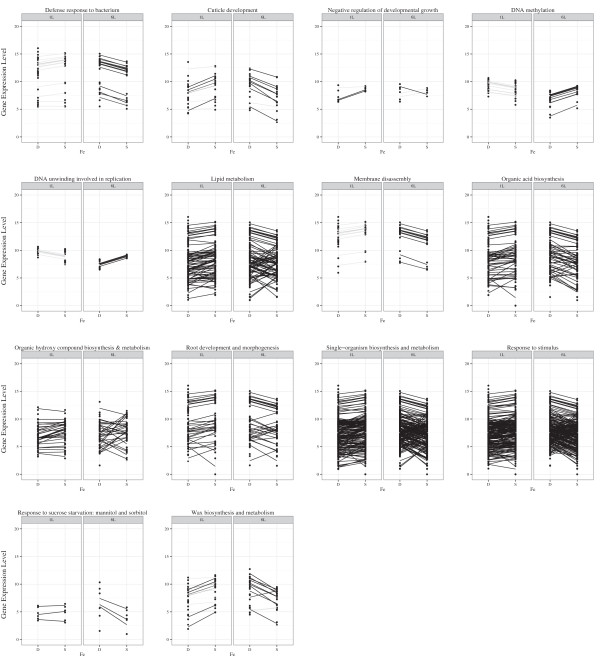
**Expression changes for genes in significantly overrepresented Biological Process GO categories in leaves.** To identify BP gene ontology terms overrepresented in our data sets, we combined all DEGs from leaves. Overrepresented gene ontology terms were identified using the Ontologizer 2.0 software [50] with parent–child-union analysis and Westfall-Young-Single-Step multiple testing correction, with a resampling of 1000 replicates. Since many of the DEGs were associated with multiple GO terms, any significant (P < 0.05) GO terms with completely overlapping DEGs were mapped to the larger (more DEGs) GO term. This data is shown in Table 2. Gene expression was plotted across time points (1 L, 1 hour leaves, 6 L, 6 hour leaves) and iron conditions (S, sufficient, D, deficient) to visualize changes. For each differentially expressed gene, both replicates are plotted with a line joining expression under deficient and sufficient conditions. The line is placed at the average of the two replicates within a condition. DEG significance within a time point is indicated by the intensity of the line.

While these analyses identified several genes of interest, they do not highlight the major plant pathways that respond to iron deficiency. Therefore, we used the Ontologizer 2.0 software [50] to identify GO terms significantly (P < 0.05) overrepresented within our DEGs, relative to all genes in the soybean genome (Table 2). In leaves, we identified twenty-eight significantly overrepresented gene ontology biological process (BP) terms. However, many of the DEGs were associated with multiple GO terms. Therefore, any significantly overrepresented GO terms whose genes completely overlapped were mapped to the largest significantly overrepresented GO term. In leaves, the twenty-eight original BP GO terms, were reduced to fourteen. Similarly, the 45 significantly overrepresented BP GO terms identified in roots were reduced to fifteen (Table 2). The fourteen significantly overrepresented BP GO terms identified in leaves included wax biosynthesis and metabolism (GO:0010025 and GO:0010166, P = 0), defense response to bacterium (GO:0009816, P = 0), and cellular response to sucrose starvation, mannitol and sorbitol (GO:0018008, GO:0018201, GO:0043617, GO:0071325, GO:0072709, P = 0.01). When gene expression patterns are compared within these GO terms across time points (Figure 2), we see opposing expression patterns. For the GO terms DNA methylation and DNA unwinding involved in replication, gene expression is induced at one hour by iron deficiency, but repressed by six hours. For all other GO terms, we see the opposite expression pattern. Further, more significant expression changes, induced and repressed, are seen at the six hour time point.

**Table 2:** **Overrepresented GO terms in leaves and roots DEGs**

Leaves description: biological processes	GO terms	DEGs	***P***-value
DNA unwinding involved in replication	GO:0006268	7	0
Membrane disassembly	GO:0030397	12	0
Wax biosynthesis and metabolism	**GO:0010025**, GO:0010166	12	0
Defense response to bacterium	GO:0009816	14	0
DNA methylation	GO:0006306	11	0
Lipid metabolism	GO:0006629	76	0
Single-organism biosynthesis and metabolism	**GO:0044710**, GO:0044711	167	0
Organic hydroxy compound biosynthesis and metabolism	GO:0006066, **GO:1901615**, GO:1901617	30	0.005
Negative regulation of developmental growth	GO:0048640	3	0.007
Response to stimulus	GO:0050896	197	0.007
Cuticle development	GO:0042335	9	0.009
Root development and morphogenesis	GO:0010101, **GO:0010015**, GO:0010311, GO:0022411, GO:0048528	30	0.01
Cellular response to sucrose starvation, mannitol and sorbitol	GO:0018008, GO:0018201, **GO:0043617**, GO:0071325, GO:0072709	3	0.012
Organic acid biosynthesis	**GO:0016053**, GO:0046394, GO:0072330	50	0.041
**Leaves description: molecular functions**			
Xyloglucan:xyloglucosyl transferase activity	GO:0016762	11	0
Transferase activity, transferring acyl groups	GO:0016746	25	0
Tetrapyrrole binding	GO:0046906	20	0.004
Oxidoreductase activity	**GO:0016491**, GO:0016661, GO:0016701, GO:0016702	59	0.029
Phosphatidylinositol binding	**GO:0005547**, GO:0043325	2	0.033
Carboxylic ester hydrolase activity	GO:0052689	18	0.033
Catalytic activity	GO:0003824	209	0.041
**Roots description: biological processes**	**GO Terms**	**DEGs**	***P*** **-value**
Cell junction organization and assembly	**GO:0034329**, GO:0034330	12	0
Root development	GO:0009913, GO:0010053, GO:0022622, **GO:0048364**, GO:0048640	40	0
Response to ethylene and other stimuli	**GO:0050896**, GO:0070887, GO:0071369	198	0
Phenylpropanoid biosynthesis and metabolism	**GO:0009698**, GO:0009699, GO:0009812, GO:0009813, GO:0009962, GO:0009963, GO:0043455, GO:1900376, GO:1900378, GO:2000762	27	0.002
Response to oxidative and other stress	**GO:0006950**, GO:0006979	155	0.002
Zinc ion transmembrane transport	**GO:0006829**, GO:0071577	6	0.003
Steroid biosynthesis and metabolism	**GO:0006694**, GO:0008202	16	0.009
Coumarin biosynthesis and metabolism	**GO:0009804**, GO:0009805	9	0.015
N-terminal peptidyl-glycine N-myristoylation	GO:0018008	2	0.021
Immune system process	GO:0002376	45	0.027
Cellular response to starvation	GO:0009267	23	0.03
Single-organism biosynthesis and metabolism	**GO:0044710**, GO:0044711	140	0.036
Cellular amine metabolic process	GO:0044106	12	0.038
Cell wall organization or biogenesis	GO:0071554	44	0.039
Cell communication	GO:0007154	85	0.044
**Roots description: molecular functions**			
Antioxidant activity	**GO:0016209**, GO:0016684	22	0
Tetrapyrrole binding	GO:0046906	26	0
Oxidoreductase activity	GO:0016491	58	0.002
Protein homodimerization activity	GO:0042803	13	0.002
Zinc ion transmembrane transporter activity	GO:0005385	6	0.002
Regulatory region nucleic acid binding	GO:0001067	32	0.003
ADP binding	GO:0043531	17	0.005
Identical protein binding	GO:0042802	16	0.005

To determine gene ontology terms overrepresented among differentially expressed genes in leaves or roots, Ontologizer 2.0 software [50] was used with parent–child-union analysis and Westfall-Young-Single-Step multiple testing correction, with a resampling of 1000 replicates. GO terms were combined when Glyma IDs overlapped entirely between two or more terms. The term containing the largest number of genes is in bold, with its corresponding P-value reported.

**Figure 3:**
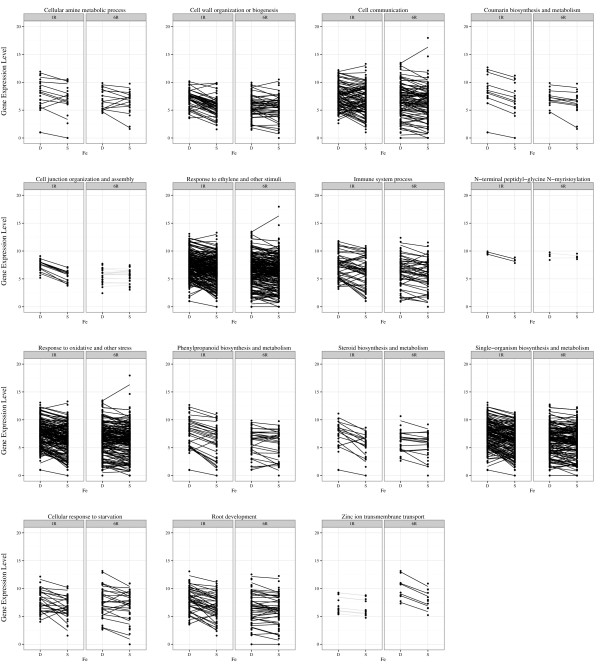
**Expression changes for genes in significantly overrepresented Biological Process GO categories in roots.** To identify BP gene ontology terms overrepresented in our data sets, we combined all DEGs from roots. Overrepresented gene ontology terms were identified using the Ontologizer 2.0 software [50] with parent–child-union analysis and Westfall-Young-Single-Step multiple testing correction, with a resampling of 1000 replicates. Since many of the DEGs were associated with multiple GO terms, any significant (P < 0.05) GO terms with completely overlapping DEGs were mapped to the larger (more DEGs) GO term. This data is shown in Table 2. Gene expression was plotted across time points (1R, 1 hour roots, 6R, 6 hour roots) and iron conditions (S, sufficient, D, deficient) to visualize changes. For each differentially expressed gene, both replicates are plotted with a line joining expression under deficient and sufficient conditions. The line is placed at the average of the two replicates within a condition. DEG significance within a time point is indicated by the intensity of the line.

The fifteen significantly overrepresented BP GO terms identified in roots included response to oxidative stress and other stress (GO:0006950 and GO:0006979, P = 0.002), zinc ion transmembrane transport (GO:0006829 and GO:0071577, P = 0.003), response to ethylene and other stimuli (GO:0050896, GO:0070887 and GO:0071369, P = 0), cellular response to starvation (GO:0009267, P = 0.03), phenylpropanoid biosynthesis and metabolism (GO:0009698, GO:0009699, GO:0009812, GO:0009813, GO:0009962, GO:0009963, GO:0043455, GO:1900376, GO:1900378, GO:2000762, P = 0.002), immune system process (GO:0002376, P = 0.027) and steroid biosynthesis and metabolism (GO:0006694 and GO:0008202, P = 0.009) (Table 2). Comparing gene expression within these GO terms at one hour and six hour roots, again the most striking observation is the direction of expression changes (Figure 3). While most leaf DEGs were repressed at one hour in response to iron deficiency, most root DEGs are induced at one hour in response to deficiency. By six hours, the expression pattern has begun to reverse. It is also worth noting the overrepresented molecular function terms identified in roots included zinc ion transmembrane transporter activity (GO:0005385), suggesting metal ion transport has been activated in the root. In addition, protein homodimerization activity (GO:0042803), regulatory region nucleic acid binding (GO:0001067) and identical protein binding (GO:0042802) suggest a strong signaling component in root responses to IDC.

When we compared our lists of DEGs to known iron homeostasis genes in *A. thaliana* [51], many were found differentially expressed in both leaves and roots (Table 3). This serves as both a control of our hydroponic iron conditions and demonstrates how quickly soybean responds to reduced iron by increasing gene expression for iron uptake and mobilization throughout the plant. While few of these genes exhibit large fold changes (>5), their function and the short response time is noteworthy. In one hour leaves, a homolog of the *AtFIT1* transcription factor (Glyma09g41470, FC = -3.8) and a homolog of the phosphate transporter PHT3;1 (Glyma01g02950, FC = -2.5), were repressed by iron deficiency. By six hours, there were equal numbers of genes with homology to known iron homeostasis genes in leaves that were induced or repressed by iron deficiency. The induced genes included an ortholog of the iron transporter *AtNRAMP3* (Glyma17g18010, FC = 4.2) and two *AtBTS* homologs, Glyma09g18770 and Glyma07g10400 (FC = 3.3 and 2.5, respectively). Only one of the four *FIT1* homologs was induced (Glyma16g02320, FC = 9.2), while a homolog of Yellow Stripe-like7 (*YSL7*) was repressed (Glyma16g33840, FC = -2.6). In roots, the response seems to initially favor an increase in transcription, and then as the iron stress persists, transport molecules such as *IRT1* and *VIT1* are upregulated, presumably to scavenge as much iron as possible to restore/maintain homeostasis. In one hour roots, all but one of the differentially expressed iron homeostasis genes identified were induced by iron deficiency. These included a homolog of the transcription factors *AtFIT1* (Glyma01g15930, FC = 3.7), an ortholog of the transcription factor *AtBHLH038* (Glyma03g28630, FC = 2.5), ferric reduction oxidases *AtFRO2* (Glyma16g03770, FC = 2.8) and *AtFRO6* (Glyma05g00420, FC = 2.8), nicotianamine synthase *AtNAS2* (Glyma08g18710, FC = 2.8) and the iron transporter *AtVIT1* (Glyma11g08830, FC = 4.0). Glyma03g28630 was recently identified as a candidate gene underlying the IDC QTL on soybean chromosome 3 [14]. In six hour roots, the homologs of the transporters *AtIRT1* (Glyma06g05460, Glyma04g05410, Glyma08g17530, Glyma13g10791, Glyma15g41620 and Glyma20g06210) and an ortholog of *AtNAS2* (Glyma19g41630, FC = 2.6) were induced by iron deficiency, while homologs of *AtVIT1* were repressed (Glyma08g08090, FC = -4.3 and Glyma05g24980, FC = -3.2).

**Table 3:** **Homologs of** ***A. thaliana*** **iron homeostasis genes found to be significantly differentially expressed**

Soybean Gene ID	Arabidopsis Gene ID	Gene function	Fold change	E-value
**One Hour Leaf**				
Glyma09g41470	AT2G28160	FIT1, BHLH029	-3.84	1.00E-06
Glyma01g02950	AT5G14040	PHT3;1 | phosphate transporter 3;1	-2.53	8.00E-14
**Six Hour Leaf**				
Glyma09g18770	AT3G18290	EMB2454, BTS | zinc finger protein-related	3.28	0.0E + 00
Glyma07g10400	AT3G18290^a^	EMB2454, BTS | zinc finger protein-related	2.48	0.0E + 00
Glyma02g42780	AT2G28160	FIT1, BHLH029	-2.48	1.00E-07
Glyma14g05870	AT2G28160	FIT1, BHLH029	-2.77	4.00E-07
Glyma01g32130	AT2G28160	FIT1, BHLH029	-5.04	6.00E-08
Glyma16g02320	AT2G28160	FIT1, BHLH029	9.24	7.00E-13
Glyma17g12450	AT5G14040	PHT3;1 | phosphate transporter 3;1	2.20	8.00E-14
Glyma17g18010	AT2G23150^a^	NRAMP3, ATNRAMP3	4.20	0.0E + 00
Glyma16g33840	AT1G65730	YSL7 | YELLOW STRIPE like 7	-2.56	0.0E + 00
**One Hour Root**				
Glyma02g14350	AT3G60330	AHA7, HA7| H(+) -ATPase 7	-2.22	4.00E-07
Glyma18g38650	AT5G62670	AHA11, HA11 | H(+)-ATPase 11	4.22	5.0E-86
Glyma05g00420	AT5G49730	ATFRO6, FRO6 | ferric reduction oxidase	2.78	9.00E-23
Glyma08g18710	AT5G56080^a^	ATNAS2, NAS2 | nicotianamine synthase 2	2.78	1.0E-89
Glyma03g28630	AT3G56970^a^	BHLH038, ORG2 | basic helix-loop-helix	2.46	5.0E-52
Glyma01g15930	AT2G28160	FIT1, BHLH029	3.68	5.00E-09
Glyma16g03770	AT1G01580^a^	FRO2, FRD1, ATFRO2 | ferric reduction oxidase	2.78	0.0E + 00
Glyma11g08830	AT2G01770	VIT1, ATVIT1 | vacuolar iron transporter	3.96	2.00E-10
**Six Hour Root**				
Glyma19g41630	AT5G56080^a^	ATNAS2, NAS2 | nicotianamine synthase 2	2.64	3.0E-136
Glyma01g02251	AT2G28160	FIT1, BHLH029	2.24	1.00E-15
Glyma06g05460	AT4G19690	IRT1 | iron-regulated transporter 1	2.99	2.00E-47
Glyma04g05410	AT4G19690	IRT1 | iron-regulated transporter 1	2.83	6.00E-63
Glyma08g17530	AT4G19690^a^	IRT1 | iron-regulated transporter 1	2.72	7.00E-99
Glyma13g10791	AT4G19690	IRT1 | iron-regulated transporter 1	5.55	4.00E-102
Glyma15g41620	AT4G19690	IRT1 | iron-regulated transporter 1	2.93	9.00E-99
Glyma20g06210	AT4G19690^a^	IRT1 | iron-regulated transporter 1	4.10	2.00E-101
Glyma08g08090	AT2G01770	VIT1, ATVIT1 | vacuolar iron transporter	-4.33	7.00E-11
Glyma05g24980	AT2G01770	VIT1, ATVIT1 | vacuolar iron transporter	-3.16	3.00E-09

Sequences of *A. thaliana* proteins identified as involved in iron homeostasis in Arabidopsis by Kobayashi and Nishizawa [51] were downloaded from The Arabidopsis Information Resource (TAIR). BLASTP (E < 10^-6^) was used to compare the protein sequence of DEGs identified in this study against the known *A. thaliana* iron homeostasis gene protein sequences. Homologous iron homeostasis genes are shown in table, divided by time and tissue. A positive fold change indicates induction in response to iron deficiency while a negative fold change indicates repression due to iron stress. ^a^Orthology between Arabidopsis and soybean proteins were verified by reciprocal best BLASTP.

### Identification of iron responsive gene families

In order to identify gene families responding to iron stress that may not have been identified in the previous analyses, we used BLASTP (E < 10^-10^) [52] and single linkage clustering [53] to group all differentially expressed genes. Using this approach, we identified 161 gene families containing 2 to 38 unique sequences (Additional file 6). Eleven gene families were identified with ten or more sequences (Groups 15, 26, 29, 34, 40, 42, 46, 53, 59, 70, 81). The majority of these gene families had largely tissue-specific expression patterns and reflected the tissues in which the largest number of DEGs were identified (one hour roots and six hour leaves). Groups 26, 59, 70 and 81 were largely specific to six hour leaves and had homology to GDSL lipases, protein kinases, cytochrome P450s and xyloglucan endotransglucosylases, respectively. Groups 15, 34, 41, 42, 46, and 53 were largely specific to one hour roots and had homology to Casparian strip membrane proteins, AP2/ERF transcription factors, peroxidases, 2-oxoglutarate (2OG) and Fe(II)-dependent oxygenases, nucleotide binding site-leucine rich repeat resistance gene homologs and dirigent-like proteins, respectively.

### Identification of transcription factors responding to iron stress

**Figure 4:**
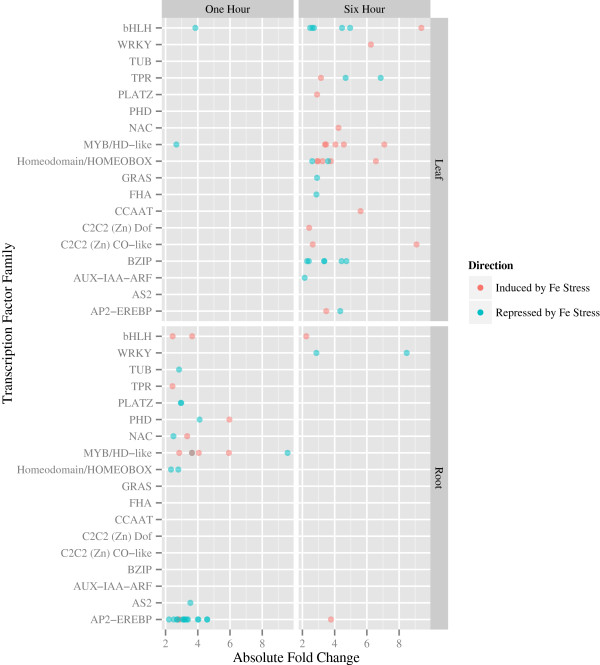
**Expression patterns of transcription factors significantly (FDR < 0.05) differentially expressed between iron sufficient and deficient conditions.** Transcription factor families on the y-axis refer to the SoyDB transcription factor database [54] as described in the methods. Absolute fold change in gene expression is plotted on the x-axis. Multiple differentially expressed transcription factors per family are plotted. Additional details on TF expression are provided in Additional file 7: Table S7.

The reversals in gene expression found between the one and six hour time points in each tissue and the overrepresentation of GO category “regulatory region nucleic acid binding” (GO:0001067, Table 2) suggested that transcription factors play a key role in the iron deficiency stress response. Therefore, we took advantage of the SoyDB transcription factor database [54], http://casp.rnet.missouri.edu/soydb/) to identify transcription factors within our DEGs (Figure 4, Additional file 7). In one hour leaves, we identified two differentially expressed transcription factors, Glyma09g41470 and Glyma17g10820, both significantly repressed by iron deficiency (fold changes of -3.8 and -2.7, respectively). After six hours of iron deficiency, only Glyma17g10820 was still significantly differentially expressed, however it was induced by iron deficiency (fold change of 3.5). Glyma09g41470 and Glyma17g10820, encode a β-helix-loop-helix and MYB/HD-like transcription factors, respectively. Their best homologs in Arabidopsis*,* identified by reciprocal BLASTP [52], have no known function.

In six hour leaves, we identified 39 differentially expressed transcription factors (Figure 4). These included representatives from the AP2-EREBP (2), AUX-IAA-ARF (1), β-helix-loop-helix (6), BZIP (6), C2C2 (Zn) CO-like (2), C2C2 (Zn) Dof (1), CCAAT (1), FHA (1), GRAS (1), Homeodomain/HOMEOBOX (7), MYB/HD-like (5), NAC (1), PLATZ (1), TPR (3) and WRKY (1) transcription factor families. Several of these have functions associated with defense or abiotic stress responses in Arabidopsis. Glyma06g05170 and Glyma08g14600 (fold changes of -4.35 and 3.48, respectively) are homologs of the AP2-EREBP transcription factor *AtDREB2C* (1E^-42^) (*d*ehydration-*r*esponsive *e*lement-*b*inding protein) which is induced by iron deficiency. *AtDREB2C* interacts with the BZIP transcription factor ABF2 to regulate ABA responsive gene expression [55]. Glyma16g01940 encodes a NAC transcription factor induced in response to iron deficiency (4.2-fold). Glyma16g01940 is homologous to *AtNTL9* (2E^-40^), which regulates osmotic stress signaling [56]. Glyma02g42380 encodes a MYB transcription factor related to *AtMYB73*, which negatively regulates SOS induction during salt stress [57].

In one hour roots, we identified 35 differentially expressed transcription factors (Figure 4). These included representative members from eight different families such as AP2-EREBP (16), AS2 (1), β-helix-loop-helix (2), Homeodomain/HOMEOBOX (2), MYB/HD-like (6), NAC (2), PHD (2), PLATZ (2), TPR (1), and TUB (1). Of the 16 AP2-EREBP proteins expressed, five are homologs of ERF1 (Glyma19g34696 [2E^-54^], Glyma20g34570 [2E^-71^], Glyma13g18410 [9E^-52^], Glyma10g33060 [1E^-73^], and Glyma11g03910, [9E^-52^]). ERF1 has been shown to regulate abiotic stress responses in Arabidopsis [58]. Glyma13g34920 encodes an ERF4 homolog (3E^-47^), which functions as a repressor in ethylene, jasmonic acid, and abscisic acid pathways [59]. Glyma03g28630 encodes a homolog of the β-helix-loop-helix transcription factor *βHLH038* (5E^-52^). This gene is upregulated under soybean iron stress conditions, and is thought to function as a binding partner for FIT [14]. Glyma16g01911 is most similar to *AtNTL6* (6E^-53^), which is a plant-specific NAC that is phosphorylated by SnRK2.8 in drought-resistance [60]. The PHD family member, Glyma20g01070, is upregulated 6-fold in response to iron deficiency and is most similar to *AtXLG2* (extra-large G protein 2, 1E^-96^), a GTP-binding protein that has been implicated in resistance to *Pseudomonas syringae* [61].

In six hour roots, we identified four differentially expressed transcription factors. Two were upregulated in response to iron stress, Glyma03g31940 and Glyma01g02251 (fold changes of 3.8 and 2.2, respectively), and two downregulated, Glyma18g10324 and Glyma08g43258 (fold changes of -8.5 and -2.9, respectively) (Figure 4). Only Glyma03g31940 was also significantly expressed at one hour, downregulated -3.4 fold at that time. Glyma03g31940 encodes an AP2-EREBP transcription factor (8E^-54^) homologous to *AtERF15*. Glyma01g02251 encodes a MYC2 homolog (E = 0), which is activated by the jasmonic acid signaling pathway to negatively regulate JA-mediated stress responses [62]. Glyma18g10324 and Glyma08g43258 each encode homologs of the *AtWRKY6* transcription factor (1E^-88^ and 2E^-88^, respectively). *AtWRKY6* has been implicated in senescence and defense [63], phosphate stress [64] and boron deficiency [65].

In addition to identifying individual transcription factors within each sample, we used overrepresentation analysis to identify transcription factor families significantly overrepresented among DEGs relative to their abundance in the soybean genome. Only the AP2-EREB transcription factor family was identified as significantly overrepresented (P < 6.88E^-05^) and only in one hour roots.

### Identification of transcription factor binding sites overrepresented in iron-responsive genes

Transcriptional cascades happen quickly, and to explore pathway components outside of our one and six hour windows, we examined the transcription factor binding sites that were overrepresented in the promoters of our DEGs. We leveraged Clover (*C*is e*l*ement *over* representation) [66] and the TRANSFAC transcription factor database (version 2010, [67]) to identify transcription factor binding sites significantly (t < 0.05) overrepresented in promoters of DEGs relative to promoters of all predicted genes in the soybean genome.

We found 74 unique transcription factor motifs significantly overrepresented across the four tissue/time points within the DEGs. Focusing on transcription factors known to be involved in abiotic and biotic stress response, ARF, BZR1, DREB1B, HY5, MYBPH3, TGA1, and TRAB1 binding sites were all significantly overrepresented (t < 0.05) in promoters of genes differentially expressed in one hour leaves (Additional file 8). ARF (auxin response factor) has been implicated in both biotic and abiotic stress responses in several plant systems [68, 69]. In our dataset, its binding site is present in a high percentage of DEG promoters (one hour leaf, 74%; six hour leaf, 70%; one hour root, 69.2%). BZR1 is a central regulator of brassinosteroid (BR) signaling, synthesis and growth responses [70]. Soybean *GmDREBa* and *GmDREBb* are induced by cold, drought and salt in the leaves of seedlings. While expression of *GmDREBc* is low in leaves, it has high levels of expression in roots following drought, salt and ABA treatments [71]. HY5 (LONG HYPOCOTYL 5) is a bZIP transcription factor that has been shown to positively regulate anthocyanin biosynthesis [72]. The MYBPH3 transcription factor functions in the regulation of flavonoid biosynthesis in petunia [73] and may also be involved in salt and cold-tolerance in pea [74]. TGA1 is controlled by nitric oxide and regulates systemic acquired resistance in plants through salicylic acid (SA)–mediated signal transduction pathway [75, 76]. TRAB1, responsible for ABA regulation, is phosphorylated in response to osmotic stress and by the SnRK2 kinase in response to ABA [77].

In six hour leaves, ABF1, ABZ1, Alfin1, ARF, AtMYB15, AtMYB77, BZR1, C1, DREB1B, E2F, HY5, KNOX3, LIM1, MYBAS1, NAC6, OSBZ8, P, RAV1, TGA1 and TRAB1 binding sites were significantly (t < 0.05) overrepresented in the promoters of our DE genes and all have reported roles in stress responses (Additional file 9). ABF (ABA-responsive elements binding factor) is ABA and stress inducible, and in turn, activates ABREs (ABA-responsive elements) in response to abiotic stress [78]. ABZ1 (anaerobic basic leucine zipper) was isolated from a tomato cDNA library enriched for anaerobically induced genes [79]. The soybean genome contains six genes identified as Alfin1-type PHD finger protein and their expression responds differentially to drought, salt, cold and ABA treatments when expressed transgenically in Arabidopsis [80]. Like ARF, Alfin binding sites are highly represented in our DEG promoters (six hour leaves, 74.3%; one hour roots, 70.6%). Myb factors have been implicated in a variety of biotic and abiotic stress responses [81]. Transgenic *AtMYB15* can confer improved tolerance to drought and salt stress in Arabidopsis [82]. *AtMYB77* expression responds to wounding, pathogen infection, abiotic stress and hormone treatment [68]. The E2F transcription factor regulates the cell cycle and DNA replication [83, 84]. Atwood *et al.* [11] and O’Rourke *et al.* [13] found that DNA replication was inhibited in iron efficient soybean lines. HAHB4, a HD-Zip transcription factor, regulates crosstalk between ethylene and drought signaling in sunflower [85]. *AP*ETALA *2*/*e*thylene-*r*esponsive element binding *f*actor (AP2/ERF) family includes four major subfamilies: the AP2, RAV, ERF and DREB subfamilies and many have been shown to play a role in abiotic stress [86]. Binding sites for two of those subfamilies (RAV and DREB) were overrepresented in the promoters of genes from the six hour leaf time point. Knotted1-like homeobox (KNOX) genes are involved in plant morphogenesis, and barley KNOX3 has been shown to be regulated by the ethylene signaling pathway [87]. Lignin plays an important role in mechanical support, water transport and pathogen resistance. *NtLIM1* encodes a Pal-box binding protein involved in lignin biosynthesis [88]. Tobacco *NtMYBAS1* is involved in phenylpropanoid biosynthesis [89], which has long been known to be stress-induced [90]. Soybean *GmNAC6* is induced by both endoplasmic reticulum-stress and osmotic-stress signaling to promote cell death [91]. In rice, the bZIP class Abscisic acid Responsive Element (ABRE)-binding factor, OSBZ8 has been shown to function in ABA signaling and in salt stress [92]. The P transcription factor in maize is involved in flavonoid biosynthesis, leading to the production of a red phlobaphene pigment [93, 94]. *AtRAV1*, a RAV (*R*elated to *A*BI3/*V*P1) transcription factor family gene has been shown to positively regulate leaf senescence, and is induced in response to ethylene and methyl jasmonate [95]. DREB1B, TGA1, and TRAB1 binding sites were all overrepresented in both one hour and six hour leaves.

In our DE genes from one hour roots, ABZ1, Alfin1, AtMYB77, BZR1, HAHB4, MYBPH3, P, TGA1a, TGA1b, TGA2, TRAB1 and WRKY11 binding sites were overrepresented (Additional file 10). Of these 11 families, only CBNAC, TGA2 and WRKY11 binding sites are unique to one hour roots. Calmodulin-regulated transcription factors and NAC transcription factors in general have been show to function in both biotic and abiotic stresses [96, 97]. TGA2 is involved in salicylic acid signaling in Arabidopsis [98]. WRKY11 is a negative regulator of basal defense responses in Arabidopsis [99]. Six hour DE root genes had P, RAV1, TGA1a and TGA1b binding sites overrepresented (Additional file 11). There are 14 transcription factor family binding sites overrepresented in all four time points and tissues, and 40 overrepresented in at least two time points and tissues.

## Discussion and conclusions

A complicated molecular network exists to maintain iron homeostasis, as metals are necessary for many metabolic processes yet toxic to cells in high concentrations. The mechanisms for sensing deficiencies and interactions with general stress and defense pathways are poorly understood. Previous work demonstrated that iron deficiency is sensed in the leaves and that an unknown leaf signal regulates the expression of iron uptake genes in the root [7, 8]. In order to capture signaling between the root and shoot, and to identify genes acting early in efficient responses, we sampled leaves and roots from the same plants, one and six hours after the onset of iron stress. One of our first observations was the dynamic difference between roots and shoots. At one hour of iron stress, few genes were differentially expressed in leaves but many were already changing expression in roots. By six hours, more genes were differentially expressed in the leaves, and a massive shift was seen in the direction of gene expression in both roots and shoots. Further, there was little overlap in the DEGs found in each tissue and time point.

Stein and Waters [100] and Waters *et al.* [101] used the Arabidopsis genome array to measure gene expression in roots and rosettes (respectively) of the same plants 24 and 48 hours after iron deficiency using two different Arabidopsis ecotypes, differing in the speed of their iron deficiency response time [100]. In the faster Kas-1 ecotype, greater differential gene expression was observed in roots (1504 DEGs) than rosettes (130 DEGs). In the slower Tsu-1 ecotype, the number of DEGs was approximately equal between roots and rosettes (630 and 690, respectively). In the faster Kas-1, 40% and 31% of DEGs were expressed in both time points in roots and rosettes, respectively. In contrast, only 16% and 10% of DEGs from Tsu-1 roots and rosettes were common to both time points. Another interesting difference between ecotypes was that only Kas-1 root DEGs were significantly overrepresented with abiotic and biotic stress associated GO terms. In contrast, only Tsu-1 rosettes were overrepresented with abiotic and biotic stress GO terms. In our study, approximately equal numbers of DEGs were identified in roots and leaves, with very little overlap in time points, mirroring Tsu-1 responses [100, 101]. In our experiment however, we found most GO terms associated with abiotic stress significantly overrepresented in the root, mirroring Kas-1. Another interesting difference is that Stein and Waters [100] found that *FRO2* expression in Kas-1 significantly increased 16 hours after iron deficiency treatment, while *FRO2* expression in Tsu-1 was significant only after 48 hours. In our study, we found that soybean homologs of FIT and FRO2 were induced by iron deficiency in both one and six hour roots suggesting Clark may have a faster iron efficiency response than either of the Arabidopsis ecotypes. Differences in the timing of *FRO2* expression could also be due to different experimental protocols. While our study reduced available iron under hydroponic conditions, Stein and Waters [100] removed iron from the growing media completely.

To our knowledge, no other gene expression analyses have been performed on the early stages of iron deficiency examining root and shoot response simultaneously. Therefore, we expanded our comparisons to other iron deficiency experiments focused on single tissues. Buckhout *et al.* [102] grew Arabidopsis ecotype Landsberg erecta in Fe-sufficient hydroponic conditions, moved them into Fe-free media and collected roots 0, 0.5, 1, 6 and 24 hours later. At one hour, they identified 36 DEG (18 induced, 18 repressed). By six hours, 60 DEGs (50 induced, 10 repressed) were found. Similar to our study, very little overlap in gene expression was found between time points. However, we found greater differential gene expression in one hour roots, with 263 of 360 DEGs induced by iron deficiency. Yang *et al*. [103] grew two Arabidopsis accessions (Col-0 and C24) on agar media, switched to Fe-deficient agar and collected roots three days later for microarray analysis. Their goal was to identify core iron-stress response genes in Arabidopsis and categorize them into functional modules. They identified 130 and 44 genes upregulated and downregulated, respectively, in response to iron deficiency. All but one gene overlapped with the iron-stress responsive genes found in the Buckhout study [102].

We also examined studies done in response to other nutrient deficiencies. Hermans *et al.* [104] looked at the effect of magnesium stress on Arabidopsis roots and mature leaves 4, 8 and 28 hours after the removal of magnesium from the media. In four-hour roots, 89 of the 97 DEGs were induced by magnesium deficiency. By eight hours, 120 of 123 genes were induced and by 28 hours only 3 of 8 genes were induced by magnesium deficiency. In the leaves, 145 of 155, 104 of 106 and 286 of 410 were induced at 4, 8 and 28 hours respectively by magnesium deficiency. Their time points do not allow for direct comparison with the Clark iron response, but the pattern is reminiscent in that roots show much activity early and taper off, while leaves have larger expression changes as stress persists.

One of the aims of this study was to identify genes directly involved in the uptake and utilization of iron in soybean. A recent review by Kobayashi and Nishizawa [51] generated a comprehensive list of genes known to be involved in iron homeostasis responses in higher plants. We used this data to identify homologous sequences, responding to iron deficiency, in our RNA-Seq data. The gene expression changes are similar to what has been shown in other plants. In total, we identified 29 DEGs with homology to known iron deficiency genes. In the soybean root, we see all the components of the iron transport machinery induced; *AHA11*, *FRO2*, *FRO6*, *FIT1*, *IRT1*, *VIT1* (at one hour), and *NAS2. VIT1* functions in moving iron into vacuoles for storage [105]. The repression of *VIT1* at six hours of iron stress suggests that the roots are switching from storage to uptake and mobilization of stored iron as the stress persists.

In Arabidopsis, most of the genes involved in iron homeostasis have been found in both leaves and roots, with *AtFIT1* notably missing from leaves [106]. It is interesting that in soybean, not only are multiple copies of *FIT1* expressed in leaves, but they are differentially expressed at both one and six hours of iron stress. Yellow Stripe-like 1, 2 and 3 are well characterized in iron-transport [107, 108], while the function of *YSL7* in metal transport is only putative. We observe *YSL7* responding to iron deficiency in leaves, but no other YSL homologs were significantly affected. *AtBTS* has been shown to function in leaf iron homeostasis [109], but more studies have been conducted on its role in the roots. Strangely, we do not see BTS in our root data, but this may be due to timing of the experiment as BTS levels are elevated in Arabidopsis roots by 24 hours of iron stress [109]. Such differences between soybean and Arabidopsis in iron response are worth investigating further.

One novel approach we used to characterize iron deficiency responses in soybean was to identify gene families among our differentially expressed genes (Additional file 6). One hour roots had a number of differentially expressed gene families with ten or more sequences. Interestingly, several of these were related to the development and maintenance of Casparian strips and all were induced by iron deficiency. Group 15 contained 13 DEGs from one hour roots orthologous to Casparian strip membrane domain proteins (CASPs) 1, 3 and 5. These proteins were identified by Roppolo *et al*. [110] for their role in the development of Casparian strips. Group 53 contained nine DEGs from one hour roots with homology to dirigent-like proteins, including orthologs of ENHANCED SUBERIN 1 (ESB1). Recently, Hosmani *et al.* [111] found that ESB1 was required for the formation of Casparian strips. Group 41 contained 18 DEGs with homology to peroxidases in one hour roots. Lee *et al.* [112] found that peroxidase *AtPER64* was also required for timely formation of Casparian strips. Other peroxidases (*AtPER03*, *AtPER09*, *AtPER15*, *AtPER37*, *AtPER39*, *AtPER40*, *AtPER49*, *AtPER72*) were also induced in the root endodermis relative to the rest of the root. Given these results we examined the rest of the differentially expressed genes for other genes that could function in Casparian strips. The top DEG in one hour root is a homolog of a type III peroxidase, RCI3 (Glyma10g02730), which has been shown to contribute to ROS production in potassium deficiency [29]. Four other homologs of RCI3 are also highly induced in one hour root (Glyma02g17060, Glyma03g36620, Glyma12g10850 and Glyma06g45910). An ortholog of respiratory burst oxide homolog F (*AtRBOHF*, Glyma05g00420) was also induced by iron stress in one hour roots. Lee *et al.* [112] found that RBOHF was also localized to Casparian strips and was required for their formation. Further, Lee *et al.* hypothesized that CASP proteins provide a scaffold for RBOHF to produced hydrogen peroxide which is then used by the peroxidases to polymerize lignins to form Casparian strips. The Casparian strip has been shown by Perls/DAB staining in *frd3* mutants to act as a barrier to Fe within the root [113]. Additionally, we see seven homologs of *NET1D* (AT1G03080; Glyma17g27187, Glyma17g23660, Glyma17g27135, Glyma13g07360, Glyma07g36351, Glyma10g14860 and Glyma15g21211) upregulated in response to iron deficiency. The NET1D family has recently been shown to be an actin-binding protein highly expressed in the stele and conducting tissues of the roots [32]. Given that Casparian strips are thought to control the passage of water and mineral nutrients into the vascular system, which would then need to pass through the stele into the xylem, it interesting to speculate that increased expression of these genes in response to iron stress could facilitate the uptake of iron.

We also identified a putative family (Group 42) of 9 2OG-Fe(II)-dependent oxygenases differentially expressed in one hour roots. While seven of these were induced by iron deficiency, two were repressed. Reciprocal best BLASTP [52] identified two orthologs of FERULOYL-COA 6’ HYDROXYLASE 1 (AtF6’H1, Glyma03g23770 and Glyma07g12210), both induced by iron deficiency. Schmid *et al.* [114] found that F6’H1 was required for coumarin synthesis and was also induced by iron deficiency. Recently, Rodríguez-Celma *et al.* [115] demonstrated that Arabidopsis excretes phenolic compounds, such as coumarin, in response to low iron. Fourcroy *et al.* [116] found that *AtPDR9* was induced by iron deficiency in Arabidopsis and was required for the secretion of coumarin compounds aiding in iron acquisition. Therefore, we examined our DEGs to identify similar genes. We found differential expression of a number of these genes in one hour roots including orthologs of pleiotrophic drug resistance protein *AtPDR9* (Glyma17g03863 and Glyma07g36166), *AtPDR11* (Glyma19g37760), *AtPDR12* (Glyma13g43860) and caffeic acid O-methyltransferase *AtOMT1* (Glyma04g40591 and Glyma06g14210). Further, we found the GO terms associated with coumarin (GO:00098040 and phenylpropanoid (GO:0009698) biosynthesis and metabolism were significantly overrepresented in one hour roots. This suggests that secretion of coumarins is essential for iron uptake in soybean as well.

The second main aim of this study was to identify signaling genes that regulate iron deficiency responses. The virtual on/off switch of gene expression we observed between one and six hours suggests a prominent role for transcription factors in establishing and regulating early iron-stress responses in roots and leaves. Our analyses of the transcription factor families within our DEG list led to interesting results. Within the 970 DEGs in this study, there were 80 transcription factors representing 18 families. We found two transcription factors, both downregulated, in one hour leaves but 35 transcription factors representing ten families that were either induced or repressed in roots at the same time. As was true for DEGs, we saw this pattern reverse by six hours with 39 transcription factors representing 15 families differentially expressed in leaves and only four transcription factors in roots.

Transcription factor binding sites, which were overrepresented in our DEGs, correlated with this pattern as well, with six hour leaves containing the highest number of unique transcription factor binding sites, and binding sites within six hour leaf transcripts greater than six hour root transcripts. Surprisingly, the degree of overlap between transcription factor family binding sites across time points and tissues was larger than might have been expected given the differences in gene expression across time points and tissues. There were 40 transcription factor binding sites that were significantly overrepresented (t < 0.05) in at least two time/tissue gene lists out of 74 transcription factor binding sites significantly overrepresented in at least one time point or tissue. Many of the transcription factor families corresponding to the significant transcription factor binding sites were not identified as significantly differentially expressed themselves, suggesting that the complexity of the early stress response is greater than what we captured.

Using a combination of different approaches, we also observed evidence of hormone-related signaling in our data. Recently, Garcia *et al.* [117] demonstrated that genes involved in iron deficiency responses, such as *AtFIT*, *AtBHLH38*, *AtFRO2*, *AtIRT1*, *AtNAS1*, *AtNAS2* and *AtFRD3,* were induced in response to IDC, ethylene, and nitric oxide (NO). Our results also confirm a role for ethylene and NO in IDC responses. In one hour roots, we observed differential expression of eleven genes involved in ethylene production, all of which were induced by iron deficiency. These encode ten oxidoreductases (Glyma02g34201, Glyma04g07480, Glyma04g07490, Glyma07g18280, Glyma08g18011, Glyma15g33740, Glyma15g41000, Glyma16g12830, Glyma18g43136 and Glyma19g31460) and a 1-amino-cyclopropane-1-carboxylate synthase (ACC, Glyma08g03400). ACC is converted to ethylene by ACC synthase and ACC oxidase [118]. While ethylene synthesis appeared to be induced by iron deficiency in one hour roots, APETALA 2/ethylene-responsive element binding protein (AP2-EREBP) transcription factors were repressed. In six hour roots, genes involved in response to ethylene (GO:0050896, GO:0070887 and GO:0071369, P=0) were significantly overrepresented and induced by iron deficiency. This included homologs of the ethylene sensors *AtEIN4* (Glyma20g21780), *AtEIN3* (Glyma17g31940), *AtERF15* (Glyma03g31940) and *AtETR2* (Glyma20g34420). Glyma03g31940 (*AtERF15*) has an extreme fold change of -3.4 in one hour root to 3.8 in six hour roots. The chitinase *AtChiB* (Glyma02g04820), PDF1.2 (Glyma03g32520) and PR4 (Glyma03g40770), which are all induced by ethylene [119, 120], were repressed in six hour roots. The promoters of genes differentially expressed in six hour roots had an overrepresentation of the AP2-EREBP DREB1B transcription factor binding site (P<1.6E^-6^). The induction of the ethylene biosynthetic enzyme 1-aminocyclopropane-1-carboxylate oxidase (ACO1, Glyma05g36310) in six hour roots, suggests that ethylene may be required for sustained IDC signaling in soybean.

NO has protective effects on iron-stressed organisms, ranging from animals to plants [121]. Homologs of proteins involved in NO synthesis and nitrate transport such as *AtNIR1* (Glyma02g14910 and Glyma07g33570), *AtNRT1* (Glyma11g03430) and *AtNIA1* (Glyma06g11430 and Glyma13g02510) were repressed by iron deficiency in one hour leaves, but homologs of the nitrate transporters *NRT1*-2 (Glyma08g407340 and Glyma08g40740) and *NRT1-7* (Glyma01g04830) were induced in six hour leaves. We also found that binding sites of the NO regulated transcription factor TGA1 [75, 76] were overrepresented in one hour leaves. These findings suggest NO synthesis is occurring mainly in the leaves. NO can interact with iron-sulfur cluster enzymes such as ribonuclease reductase, aconitase and NADH dehydrogenase to inhibit DNA synthesis and mobilize stored iron reserves. Aconitase *ACO3* (Glyma14g12315) was induced in one hour roots while NADH dehydrogenase *NAD2* (Glyma0886s50) was down in six hour roots. In Arabidopsis and cucumber, ACO1 (ACC oxidase) is involved in ethylene synthesis, has also been shown to increase NO production, and we see *ACO1* (Glyma05g36310) induced in six hour roots [9]. We also see two nitrate transporters differentially expressed, but in opposite direction in roots after six hours of iron stress; *AtNRT2*.4 (Glyma12g08380, FC=2.95) and *AtNRT1*-5 (Glyma01g40850, FC=-4.38).

We observed other interesting signaling pathways changing in response to iron deficiency including genes involved in the sucrose efflux pathway. SWEET transport proteins act redundantly to mediate sucrose efflux in Arabidopsis [23]. *atsweet11*;*12* double mutants were defective in phloem loading, had reduced growth, and had increased sucrose levels in the leaves. In addition, expression of SWEET proteins was tightly correlated with other sucrose synthesis and transport genes including sucrose phosphate synthase (*AtSPS4F*) and a sucrose transporter (*AtSUC2*). In our experiments we observed repression of two SWEET13 sucrose transporters (Glyma05g38340 and Glyma08g01310), a homolog of *AtSPS3F* (Glyma14g03300), and a homolog of *AtSPS4F* (Glyma15g03300) in the leaves one hour after iron stress. However, a homolog of *AtSWEET12* (Glyma05g38351) was induced by iron stress in one hour leaves. By six hours after iron stress, none of the soybean SWEET genes were differentially expressed but a homolog of *AtSUC2* (Glyma16g27350) was induced by iron stress. Similarly, genes involved in response to sucrose starvation were significantly overrepresented in six hour leaves (GO:0043617, P < 0.05). Alterations in sucrose efflux could signal stressful conditions from shoot to root by limiting root growth and potentially affecting nutrient uptake.

Identifying genes involved in sugar signaling ties in with previous work from our group investigating the role of DNA replication in iron deficiency stress responses. O’Rourke *et al.* [13] found that genes involved in DNA replication were repressed in leaves in response to long term (14 days) iron stress in the iron efficient line Clark. Atwood *et al.* [11] found that DNA replication genes were differentially expressed between two near isogenic lines (Clark and Isoclark) that differed in their iron efficiency. Silencing of the DNA replication gene *GmRPA3c* (Replication protein A subunit 3) in Isoclark, to mimic expression in Clark, resulted in improved IDC symptoms and significantly reduced growth. RNA-Seq of silenced plants revealed *GmRPA3c* silencing resulted in massive transcriptional reprogramming with genes involved in defense and immunity, circadian rhythm, photosynthesis, protein modifications, growth and iron uptake and transport significantly differentially expressed. We hypothesized [11] this response was controlled by the SnRK1/TOR complex which is regulated by sucrose and heavy carbon load [122]. Activation of SnRK1, initiates a phosphorylation relay [123, 124] that inhibits components of the SnRK1/TOR pathway including the E2F transcription factor that controls the cell cycle and DNA replication [83]. In the analysis reported here, we see differential expression of genes involved in sucrose transport and DNA replication. In addition, we find that E2F transcription factor binding site (M01114) is significantly overrepresented (P=0) in six hour leaves and that a homolog of *AtETG1* (*E2F TARGET GENE 1*) is repressed by iron deficiency.

In a general sense, the SnRK1/TOR signaling pathway is controlled by nutrient and energy availability inside the cell, but it remains unclear how external and endogenous signals regulate nutrient and energy status. Recently Schröder *et al.* [125–127] characterized the extracellularly located EXO and EXO-like (EXL) family in Arabidopsis. While EXO is required for growth under standard conditions, EXL1, EXL2 and EXL4 function to slow growth during low carbon availability. Lisso *et al.* [38] used *exo* T-DNA mutants and EXO overexpression in *exo* mutants coupled with sucrose and trehalose feeding studies to study the function of EXO. They found that *exo* mutants grew slowly, regardless of sugar levels, suggesting EXO modifies sugar responses during seedling growth. Further, EXO regulated the expression of a number of sugar responsive genes including *AtDIN6* and *AtSUC2*. They hypothesized that EXO could link extracellular and intracellular carbon and sugar signaling. In our six hour leaf data, we identified eight homologs of *EXO* and *EXL5* (Glyma02g37060, Glyma04g10880, Glyma06g10700, Glyma06g10710, Glyma10g32250, Glyma14g35330, Glyma14g35340 and Glyma20g35370) repressed in response to iron deficiency with fold changes ranging from -3.3 to -45.2. Similarly, three homologs of *AtDIN6* (Glyma02g39320, Glyma11g27480 and Glyma18g06840) and the homolog of *AtSUC2* mentioned above were all induced by iron deficiency. These data suggest that SWEET and EXO proteins regulate the SnRK1/TOR signaling pathway in response to iron deficiency. Further, recent work by Xiong *et al*. [128] shows that both sucrose and glucose signaling are components of the SnRK1/TOR pathway. Our data adds support to the model proposed in Atwood *et al.* [11] that iron deficiency in Clark is regulated by SnRK1/TOR signaling.

The coordination of growth and developmental pathways with stress responses makes sense, however many IDC studies have focused on long term stress responses. In soybean, even short term IDC has a long lasting effect on yield [129]. While iron efficient soybean lines would seem preferable, they generally yield lower than iron inefficient lines in iron sufficient conditions [130], again suggesting a link between the regulation of growth and development and nutrient stress. Given this response, IDC tolerant soybean lines are not employed by farmers unless completely necessary. Therefore, research needs to focus on translating expression studies to the identification of target genes for crop improvement. While our analysis identified hundreds of DEGs, identifying those genes responsible for greater stress tolerance that have little or no impact on yield is an important challenge for the future.

## Methods

### Growth conditions

Soybean (*Glycine max* (L.) Merr.) line Clark (PI 548533) was germinated on germination paper for one week at the USDA-ARS green house at Iowa State University. Seedlings were transferred to hydroponics with iron sufficient media (100 μM Fe(NO3)3•9H2O) and 3% CO2 as described by Chaney *et al*. [131], with volumes adjusted for 10 L buckets. Nine days after being placed in hydroponics, the roots of all seedlings were rinsed six times in diH_2_O and transferred to either Fe sufficient or deficient nutrient solutions (100 μM vs. 50 μM Fe(NO3)3•9H2O). Chaney *et al*. [131] demonstrated that these nutrient solutions distinguished iron efficient and inefficient cultivars and mimicked IDC symptoms in the field. These growth conditions have also been used in other soybean iron deficiency studies [11–15]. Whole roots and the 1^st^ trifoliate of plants were harvested at one hour and six hours after transfer into the separate Fe conditions and flash frozen in liquid nitrogen. Two biological replicates were harvested for each sample. As samples were collected before the onset of IDC, A15 (iron efficient) and T203 (iron inefficient) control plants were grown to verify expected IDC symptoms in Fe-deficient conditions. The tissues used in this study were the same tissues used by Atwood *et al*. [11] to study the effect of iron deficiency on DNA replication genes.

### RNA isolation

Flash frozen tissue was ground in liquid nitrogen with a mortar and pestle. RNA was extracted using a Qiagen® RNeasy® Plant Mini Kit (Qiagen®, Germantown, MD). The manufacturer’s protocol was followed using ~300 mg of ground tissue which was lysed using the RLT buffer and tubes were incubated at 56°C for two minutes with 800 rpm shaking to aid in tissue disruption. RNA was treated with an Ambion® TURBO DNA-free™ kit (Ambion®, Austin, TX) to remove all contaminating DNA. RNA quality was analyzed using an Agilent® 2100 Bioanalyzer TM (Agilent®, Santa Clara, CA). RNA was considered to be of good quality if the RNA was not degraded or was only marginally degraded. Equal amounts of RNA from three plants were pooled for each biological replicate prior to sequencing. In addition, the same RNA samples were used by Atwood *et al*. [11] to measure differential gene expression of Replication Protein A subunits by quantitative reverse transcription polymerase chain reaction.

### RNA-Seq and data analysis

Sequencing was performed at the National Center for Genome Resources on an Illumina Genome Analyzer II as described by Peiffer *et al.* [14]. In brief, 16 multiplex libraries were prepared from two biological replicates of the eight samples (one and six hour samples of roots grown in sufficient or deficient Fe conditions and leaves grown in sufficient or deficient Fe conditions). Libraries were sequenced for 36 cycles to produce a total of 507,784,149 single-end reads. TopHat (version 2.0.3, [132]) was used to align paired reads to the Williams 82 reference genome sequence using default settings (version *G. max* 1.1, [16]). The program samtools [133] was used to remove unreliably mapped reads. The resulting mapping files (bam) were imported into the statistical program R (R Development Core Team 2006) using the Bioconductor package Rsamtools [134]. The Bioconductor package rtracklayer [135] was used to import the gene feature file corresponding to *G. max* version 1.1 [16]. The package GenomicRanges [136] was used to count reads and output a matrix containing gene counts for each sample. Genes with counts per million (cpm) < 1 in at least two of the four samples being compared were eliminated from the analyses. Count tables for all genes are provided in Additional files 12, 13, 14 and 15. The Bioconductor package edgeR [17, 137–139] was used for single factor, pairwise comparisons to calculate normalization factors, estimate tagwise dispersion and determine differential expression (DE). Differential expression compared iron sufficient conditions to the iron deficient conditions (D/S). The R graphics program ggplot2 [18] was used to compare sample replicates for technical reproducibility (data not shown) and to create porcupine plots comparing gene expression of DEGs and their replicates to all other expressed genes. Data was visualized at multiple FDR (Figure 1, Additional file 1). Following visual assessment, DE genes were considered significant if their fold change was greater than two with a false discovery rate (FDR) <0.05.

### Annotation of DEGs

DEGs were annotated using the SoyBase Genome Annotation Report page (http://soybase.org/genomeannotation/index.php). In brief, primary proteins of *G. max* version 1.1 were compared to the UniRef100 protein database (version 11/26/2012, [20] and all predicted proteins from the *A. thaliana* genome (The Arabidopsis Information Resource version 10) using BLASTP (E<10^-6^, BLAST version 2.2.27, [52]. BLAST reports from Uniref100 were parsed using custom Perl scripts to identify the top BLASTP hit and the most informative BLASTP hit. Informative BLASTP hits were identified by removing hits containing the key words predicted, hypothetical, related, and scaffold. Custom Perl scripts were used to assign gene ontology (GO) biological process and molecular function terms [140] information from the top *A. thaliana* hit to the corresponding soybean protein. To identify gene ontology terms overrepresented among DEGs from each tissue, we used Ontologizer 2.0 software [50] with parent-child-union analysis and Westfall-Young-Single-Step multiple testing correction, with a resampling of 1000 replicates [141]. DEGs from each time point within a tissue were combined. The gene ontology information from Arabidopsis (described above) was used to create a gene associate file for soybean for use with Ontologizer.

In order to identify transcription factors present within the DE genes, we took advantage of the SoyDB transcription factor database [54]. However, the database had not been updated to reflect changes in *G. max* version 1.1 [16]. Best reciprocal BLASTP ([52], E<10^-6^) was used to compare all predicted proteins in *G. max 1.0* to all predicted proteins from *G. max 1.1.* Custom Perl scripts were then used to assign transcription factors in SoyDB to a *G. max* 1.1 identifier. Of the 5,683 transcription factors present in SoyDB, 5,124 (90%) were assigned *G. max* 1.1 identifiers. To identify overrepresented transcription factor families, a Fisher’s exact text [142] was used with a Bonferroni correction [143] (P<0.05) to compare the number of times each transcription factor family was found within the DEGs relative to representation in the soybean genome.

To identify soybean orthologs of known iron homeostasis genes in Arabidopsis, we leveraged the work of Kobayashi and Nishizawa [51], which identified genes involved in iron regulation, uptake and/or translocation. To identify orthologous sequences, the corresponding protein sequences were used for best reciprocal BLASTP ([52], E<10^-6^) against all predicted primary proteins in *G. max* 1.1 [16]. The Arabidopsis proteins were involved in iron regulation, uptake and/or translocation. To account for whole genome duplication events in soybeans’ evolutionary history, each Arabidopsis protein was allowed to hit two soybean proteins. Soybean proteins were considered putative orthologs if they identified the original Arabidopsis query sequence.

### Identification of differentially expressed gene families

Single linkage clustering (as described by [53]) was used identify gene families that could be acting in iron deficiency responses. In short, protein sequences corresponding to all differentially expressed genes were compared against themselves using BLASTP ([52], E < 10^-10^). Proteins with overlapping BLAST reports were assigned to groups representing potential gene families. For genes of interest, orthology to genes in Arabidopsis was confirmed using best reciprocal BLASTP ([52], E < 10^-10^).

### Transcription factor binding site analysis

Clover (*C*is e*l*ement *over* representation), [66] was used in conjunction with the TRANSFAC transcription factor database (version 2010, [67]) to identify transcription factor binding sites that were significantly (t < 0.05) overrepresented in promoters of DEGs when compared to the promoters of all predicted genes in the soybean genome. Custom Perl scripts were used to mine the soybean gene features file [16] (http://www.phytozome.net) and identify 1000 bases of promoter sequence for each predicted gene. Promoters were defined as the 1000 base pairs upstream of the start ATG. Promoters less than 1000 bases or containing two or more ambiguous bases (N) were removed from the analyses. Clover [66] was run using a t-value cutoff of t < 0.05. The promoters of all predicted proteins in the soybean genome were used to correct for oversampling.

## Electronic supplementary material

Additional file 1: **Genes significantly differentially expressed in response to iron stress at FDR < 0.01.** Significantly differentially expressed genes (DEGs) (FDR < 0.01) were identified by comparing gene expression in iron deficient conditions to iron sufficient conditions (D/S). Porcupine plots were used to visualize the expression of all genes and all DEGs. Expression of all genes is shown in grey. Expression of DEGs is shown in red (repressed by iron deficiency) and blue (induced by iron deficiency). A line joins replicates of DEGs. A. DEGs from leaves after one hour of iron stress. B. DEGs from leaves after six hours of iron stress. C. DEGs from roots after one hour of iron stress. D. DEGs from roots after six hours of iron stress. (PPTX 3 MB)

Additional file 2: **Genes significantly differentially expressed in leaves one hour post iron stress.** Glyma1.1 ID refers to *Glycine max* version 1.1 release. For more information see http://www.phytozome.net/soybean.php. The top descriptive Uniref100 BLASTP hit was determined via BLASTP [52] of Glyma1.1 primary proteins against Uniref100 (version 11/26/2012). The generated BLAST report was parsed to eliminate uninformative hits with descriptions including the words uncharacterized, putative, related, predicted, orf or expressed. Descriptions containing *Arabidopsis* or Rice gene identifiers (AtXgXXXXX, OsXXgXXXXX) were also ignored. A minimum E-value score E < 10^-6^ was required. The percent coverage determined by dividing the top high scoring pair length by predicted protein length. The top *A. thaliana* hit (TAIR version 10) was determined by BLASTP of Glyma1.1 primary proteins against *A. thaliana* proteins (TAIR10, E < 10^-6^). Gene ontology information was inferred from the top *A. thaliana* protein. (XLSX 26 KB)

Additional file 3: **Genes significantly differentially expressed in leaves six hours post iron stress.** Glyma1.1 ID refers to *Glycine max* version 1.1 release. For more information see http://www.phytozome.net/soybean.php. The top descriptive Uniref100 BLASTP hit was determined via BLASTP [52] of Glyma1.1 primary proteins against Uniref100 (version 11/26/2012). The generated BLAST report was parsed to eliminate uninformative hits with descriptions including the words uncharacterized, putative, related, predicted, orf or expressed. Descriptions containing *Arabidopsis* or Rice gene identifiers (AtXgXXXXX, OsXXgXXXXX) were also ignored. A minimum E-value score E < 10-6 was required. The percent coverage determined by dividing the top high scoring pair length by predicted protein length. The top *A. thaliana* hit (TAIR version 10) was determined by BLASTP of Glyma1.1 primary proteins against *A. thaliana* proteins (TAIR10, E < 10^-6^). Gene ontology information was inferred from the top *A. thaliana* protein. (XLSX 134 KB)

Additional file 4: **Genes significantly differentially expressed in roots one hour post iron stress.** Glyma1.1 ID refers to *Glycine max* version 1.1 release. For more information see http://www.phytozome.net/soybean.php. The top descriptive Uniref100 BLASTP hit was determined via BLASTP [52] of Glyma1.1 primary proteins against Uniref100 (version 11/26/2012). The generated BLAST report was parsed to eliminate uninformative hits with descriptions including the words uncharacterized, putative, related, predicted, orf or expressed. Descriptions containing *Arabidopsis* or Rice gene identifiers (AtXgXXXXX, OsXXgXXXXX) were also ignored. A minimum E-value score E < 10^-6^ was required. The percent coverage determined by dividing the top high scoring pair length by predicted protein length. The top *A. thaliana* hit (TAIR version 10) was determined by BLASTP of Glyma1.1 primary proteins against *A. thaliana* proteins (TAIR10, E < 10^-6^). Gene ontology information was inferred from the top *A. thaliana* protein. (XLSX 120 KB)

Additional file 5: **Genes significantly differentially expressed in roots six hours post iron stress.** Glyma1.1 ID refers to *Glycine max* version 1.1 release. For more information see http://www.phytozome.net/soybean.php. The top descriptive Uniref100 BLASTP hit was determined via BLASTP [52] of Glyma1.1 primary proteins against Uniref100 (version 11/26/2012). The generated BLAST report was parsed to eliminate uninformative hits with descriptions including the words uncharacterized, putative, related, predicted, orf or expressed. Descriptions containing *Arabidopsis* or Rice gene identifiers (AtXgXXXXX, OsXXgXXXXX) were also ignored. A minimum E-value score E < 10^-6^ was required. The percent coverage determined by dividing the top high scoring pair length by predicted protein length. The top *A. thaliana* hit (TAIR version 10) was determined by BLASTP of Glyma1.1 primary proteins against *A. thaliana* proteins (TAIR10, E < 10^-6^). Gene ontology information was inferred from the top *A. thaliana* protein. (XLSX 74 KB)

Additional file 6: **Gene families identified among DEGs.** Protein sequences corresponding to all differentially expressed genes were compared to each other using BLASTP ([52], E < 10^-10^). Single linkage clustering [53] was used to identify groups of proteins with overlapping BLAST reports representing potential gene families. (XLSX 127 KB)

Additional file 7: **Significantly differentially expressed transcription factors.** (XLSX 49 KB)

Additional file 8: **Significantly overrepresented transcription factor binding sites within one hour leaves DEGs.** (XLSX 50 KB)

Additional file 9: **Significantly overrepresented transcription factor binding sites within six hour leaves DEGs.** (XLSX 51 KB)

Additional file 10: **Significantly overrepresented transcription factor binding sites within one hour roots DEGs.** (XLSX 53 KB)

Additional file 11: **Significantly overrepresented transcription factor binding sites within six hour roots DEGs.** (XLSX 50 KB)

Additional file 12: **Normalized gene count table for one hour leaf samples.** The following abbreviations are used: C Clark, L leaf, D deficient Fe condition, S sufficient Fe condition, A Sample A and B Sample B. (XLSX 3 MB)

Additional file 13: **Normalized gene count table for six hour leaf samples.** The following abbreviations are used: C Clark, L leaf, D deficient Fe condition, S sufficient Fe condition, A Sample A and B Sample B. (XLSX 3 MB)

Additional file 14: **Normalized gene count table for one hour root samples.** The following abbreviations are used: C Clark, R root, D deficient Fe condition, S sufficient Fe condition, A Sample A and B Sample B. (XLSX 3 MB)

Additional file 15: **Normalized gene count table for six hour root samples.** The following abbreviations are used: C Clark, R root, D deficient Fe condition, S sufficient Fe condition, A Sample A and B Sample B. (XLSX 3 MB)
