# Supplementary material for: Identification of candidate genes involved in early iron deficiency chlorosis signaling in soybean (Glycine max) roots and leaves
Source: BMC Genomics. 2014 Aug 22;15:702. doi: 10.1186/1471-2164-15-702 (PMC4161901; doi:10.1186/1471-2164-15-702)
Supplement: Supplementary file 1 — Additional file 1: Genes significantly differentially expressed in response to iron stress at FDR < 0.01. Significantly differentially expressed genes (DEGs) (FDR < 0.01) were identified by comparing gene expression in iron deficient conditions to iron sufficient conditions (D/S). Porcupine plots were used to visualize the expression of all genes and all DEGs. Expression of all genes is shown in grey. Expression of DEGs is shown in red (repressed by iron deficiency) and blue (induced by iron deficiency). A line joins replicates of DEGs. A. DEGs from leaves after one hour of iron stress. B. DEGs from leaves after six hours of iron stress. C. DEGs from roots after one hour of iron stress. D. DEGs from roots after six hours of iron stress. (PPTX 3 MB) [file 12864_2014_6420_MOESM1_ESM.pptx]

## Slide 1
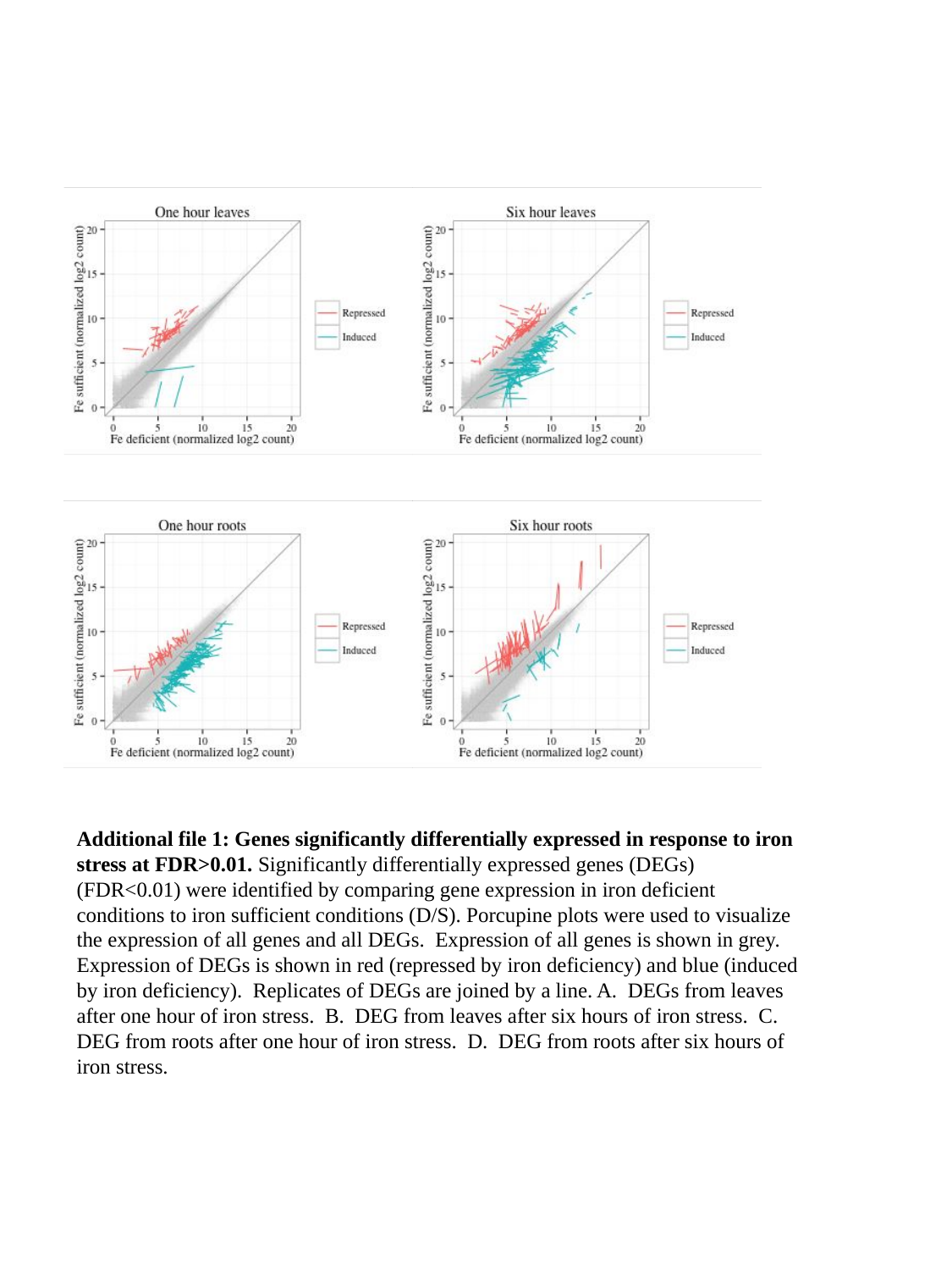

# Additional file 1: Genes significantly differentially expressed in response to iron stress at FDR>0.01. Significantly differentially expressed genes (DEGs) (FDR<0.01) were identified by comparing gene expression in iron deficient conditions to iron sufficient conditions (D/S). Porcupine plots were used to visualize the expression of all genes and all DEGs. Expression of all genes is shown in grey. Expression of DEGs is shown in red (repressed by iron deficiency) and blue (induced by iron deficiency). Replicates of DEGs are joined by a line. A. DEGs from leaves after one hour of iron stress. B. DEG from leaves after six hours of iron stress. C. DEG from roots after one hour of iron stress. D. DEG from roots after six hours of iron stress.
